# Welfare in Nile Tilapia Production: Dorsal Fin Erection as a Visual Indicator for Insensibility

**DOI:** 10.3390/ani11103007

**Published:** 2021-10-19

**Authors:** Bruno Camargo-dos-Santos, Clarissa Lerois Carlos, João Favero-Neto, Nina Pacheco Capelini Alves, Bruno Bastos Gonçalves, Percília Cardoso Giaquinto

**Affiliations:** 1 Structural and Functional Biology Department, Institute of Biosciences of Botucatu, São Paulo State University, Botucatu 18618-689, SP, Brazil; bruno.camargo@unesp.br (B.C.-d.-S.); clarissalerois88@gmail.com (C.L.C.); faverojn@gmail.com (J.F.-N.); nnpca00@gmail.com (N.P.C.A.); 2 Aquaculture Center of Unesp, São Paulo State University (UNESP), Jaboticabal 14884-900, SP, Brazil; 3 Laboratory of Environmental Biotechnology and Ecotoxicology, Institute of Biosciences of UFG, Goiás Federal University, Goiania 74690-900, GO, Brazil; goncalves.b.b@gmail.com

**Keywords:** animal welfare, aquaculture, sensibility state, pre-slaughter, fish stunning

## Abstract

**Simple Summary:**

Aquaculture has been the fastest-growing production segment in recent years, and as such, it is necessary to have clear guidelines on how fish are reared, stunned and slaughtered, always taking into account their welfare. By aiming for the stunning stage to be efficient, quick and practical before the fish are finally slaughtered in the fish farming process, we propose to verify and validate dorsal fin erection as a painless visual indicator of sensibility in Nile tilapia, ensuring that animals do not suffer during the stunning and slaughtering processes. Our results have validated the method as an effective indicator to assess the state of fish sensibility, and is simple to be carried out in large-scale production systems. The presence/absence of an erect dorsal fin alone does not totally ensure fish insensibility, and must be used together with other well-established visual sensibility indicators, for a better assessment of the state of fish sensibility, such as fish equilibrium, vestibulo-ocular reflex and opercular beats.

**Abstract:**

In aquaculture, to ensure animal welfare in pre-slaughter and slaughter stages, it is fundamental that fish are insensible. A method for evaluating fish insensibility is based on visual sensibility indicators (VSI) assessment (i.e., self-initiated behavior, responses to stimuli and reflexes). However, many stimuli used to assess fish responses are painful. Therefore, this study verifies whether the presence/absence of a dorsal fin erection (DFE) response can be used as a painless VSI in Nile tilapia (*Oreochromis niloticus*). Three stunning protocols were applied to fish: benzocaine anesthesia (40 mg/L and 80 mg/L), ice water immersion (0–1, 2–3 and 5–6 °C) and CO_2_ stunning. After these stunning methods were applied in fish, the time of loss and return of DFE was observed, along with the vestibulo-ocular reflex (VOR). All fish stunned using benzocaine and ice water immersion lose both VSIs, while 95% of fish stunned using CO_2_ lose these VSIs. In all treatments, DFEs return quicker than VOR. Therefore, DFE can be used as a VSI in Nile tilapia, which is simple for producers to assess and does not require a painful stimulus. However, the DFE alone does not totally ensure fish insensibility and must be used together with other well-established VSIs at fish farms.

## 1. Introduction

Aquaculture represents one of the major meat production systems for human consumption [1]. The accelerated growth and the high levels of production each year has been attracting the world’s attention to this segment, which is not exempt from criticism and pressure from the industry, consumers, interest groups and authorities [2,3,4,5]. More recently, the public is increasingly more aware of the importance of animal welfare and the existence of sentience in fish [6,7,8,9]. This current attitude is changing the traditional production model that has been used for years [10]. Therefore, it is necessary to have well-established protocols with clear guidelines that ensure fish welfare in all farming stages, from rearing the fish to slaughtering them [11].

In the last stage of production, slaughtering, it is well-established that for good welfare, fish must be stunned before killing, thus avoiding any pain and suffering [12,13]. Stunning methods that immediately cause a loss of sensibility and/or the consciousness of fish are considered humane and are the most recommended, such as electrical and mechanical stunning [12]. However, with electrical stunning, it is difficult to standardize and implement because there must be adequate intensity, frequency and duration for each species to cause immediate insensibility or killing. Moreover, for electrical stunning to be successful, it depends on several factors such as animal size, species, the position of the animal the tank and number of individuals per procedure [12]. This stunning method generally induces a short period of insensibility; as such, the chosen killing method must be applied quickly after stunning the fish to ensure that they die before sensibility returns [13]. Regarding mechanical stunning protocols, such as percussive stunning, spiking coring and free bullet, they result in both stunning and killing simultaneously. These methods also have some disadvantages; for example, they are applicable to a limited number of fish and require precision in the execution, since the animals usually are agitated and move in a disorderly manner. If they are not stunned correctly, it may cause partial insensibility and/or injuries to the animals [12].

Even with humane stunning methods, which are the most recommended for commercial fish slaughtering, other stunning protocols that do not immediately cause fish insensibility are still widely used worldwide given their practicality in being applied in large scale productions and since they are long lasting, such as the carbon dioxide (CO_2_) stunning in several Europe countries [5,13,14] and immersion in ice water in Brazil [15]. With CO_2_ stunning, water acidification caused by CO_2_ saturation is stressful to fish and causes aversive reactions, such as vigorous attempts to escape, that can sometimes result in damaging the fish as they hit other fish or the sides of the tank [16]. Regarding immersion in ice water, this is usually recommended for warm water species, and besides being used as a stunning method, it is also used as a killing procedure [13,16]. However, the time taken until the fish become insensible and/or die can be prolonged and stressful, increasing fish plasma cortisol levels and heart rate, as well causing averse behavior in some species until reaching insensibility or death [13]. Therefore, these methods are controversial, since they cause unnecessary prolonged suffering and stress until the fish are stunned [12,16,17]. As such, whether fish insensibility is gradually induced, it must be as fast as possible to avoid prolonged stress, suffering and other negative states [18,19].

Therefore, it is important to determine how fast a stunning protocol can induce fish insensibility, which could be a difficult task in practice. Electroencephalographic (EEG) methods provide reliable measurements about the brain function in animals, allowing the sensibility state in individuals to be measured in many farm animals, including fish [13,19]. However, an EEG can be an invasive method, demanding time, and as such, it is difficult to be used in fish production [18]. Thus, a field protocol to assess fish brain function before slaughter was developed, and so, any pain and suffering was avoided in this stage of production [18]. This protocol is based on the observation of the presence/absence of self-initiated behavior, responses to stimuli and reflexes. Clinical reflexes mediated by the brainstem (e.g., the vestibulo-ocular reflex—VOR) are extensively accepted to assess brain function in many animals, including fish [18,20]. Regarding self-initiated behavior and responses to stimuli, whether fish are able to detect and react to external events, they retain sufficient brain function to perceive strong and painful external stimulus, such as the slaughter [18]. However, all the behavioral responses used to assess the state of sensibility in fish depend on a painful stimulus, such as a tail pinch, prick or shock on a lip [11,18]. Therefore, behavioral responses that allow us to assess the state of sensibility in fish that do not depend on a painful stimulus must be investigated and incorporated into protocols that have been developed to assess fish insensibility before slaughter [11,18], avoiding any unnecessary pain and suffering in this procedure.

A behavioral response to a tactile stimulus present in many fish species is the dorsal fin erection (DFE) [21]. The DFE is a defensive behavior in some species, since extending fish spines may hurt a predator or act as a threat [22,23]. As such, it is expected that any possible threat to fish, such as quick and low-intensity handling, can set off this behavioral response, even for a painless handling. Thus, the aim of this study is to verify whether the presence/absence of a DFE response can be used as a visual indicator for the state of sensibility of Nile tilapia (*Oreochromis niloticus*) submitted to different stunning protocols. The Nile tilapia is an African cichlid, which presents DFE behavior in aggressive displays during hierarchical and territorial confrontations [23,24] and when a threat or predation risk is perceived [23]. In addition, the Nile tilapia is the third most-farmed fish species globally [1] and has a welfare assessment protocol for production systems, including the assessment of the state of fish sensibility pre-slaughter using visual indicators [11]. As such, the continuous refinement of welfare protocols is important for a better assessment of the welfare of Nile tilapia during all the production stages.

## 2. Materials and Methods

### 2.1. Fish and Rearing Conditions

We used a total of 140 Nile Tilapia from a stock population obtained from a commercial fish farm. Fish were kept in a 1200 L tank (6 L fish^−1^) during the experiment, with an average temperature of 25 ± 1 °C. The tank was supplied with continuously aerated, constantly dechlorinated water, with biological filters and thermostats. To maintain the physical-chemical water parameters, the tank was cleaned four times a week. The pH level was kept at 7 and ammonia and nitrite levels at <0.5 ppm and <0.05 ppm, respectively. The photoperiod was 12 h of light and 12 h of dark, and fish were fed once daily with commercial food (Presence Nutripiscis Si Crescimento, 28% protein). Fish used in experiment had an average weight of 42.9 ± 12.99 g and size of 13.85 ± 1.39 cm. All procedures used in this project were approved by the CEUA (Committee on Ethics in the Use of Animals) of São Paulo State University (UNESP), protocol #678/2014.

### 2.2. Experimental Design and Procedures

**Figure 1:**
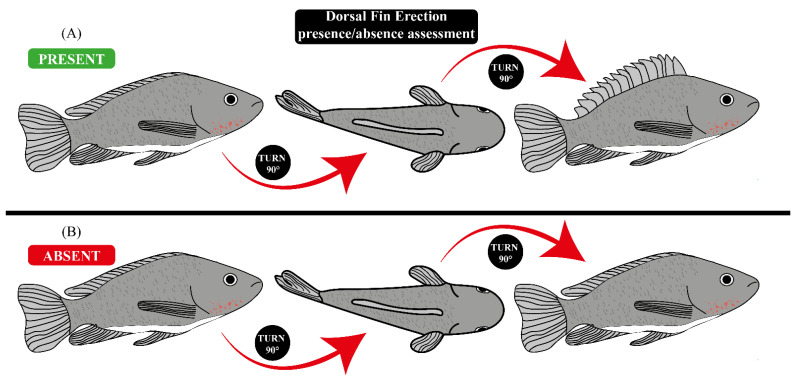
The handling needed to check whether the dorsal fin erection response is present or absent. (**A**) Dorsal fin erection present in a fish after handling. (**B**) Dorsal fin erection absent in a fish after handling.

**Figure 2:**
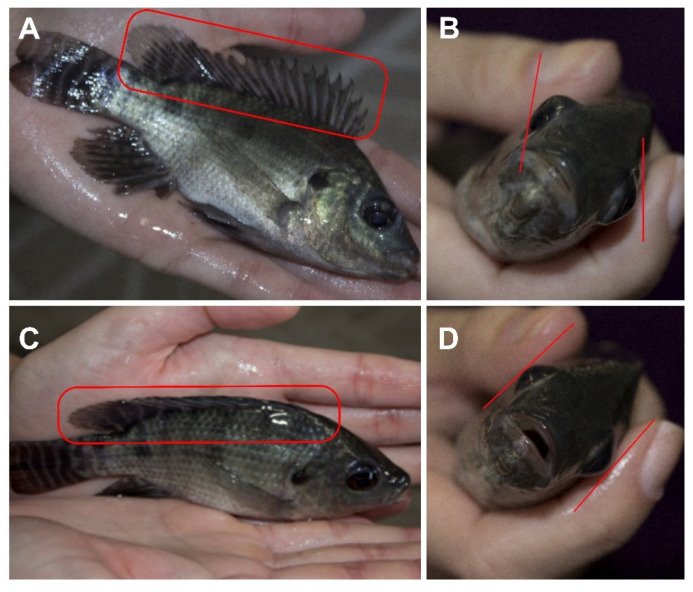
Presence and absence of responses and reflexes. (**A**) Presence of dorsal fin erection (DFE) indicated by the red rectangle and (**B**) presence of vestibulo-ocular reflex (VOR) indicated by the red line (“eye roll”). (**C**) Absence of DFE indicated by the red rectangle and (**D**) absence of VOR (“eye roll”) indicated by the red line.

Initially, we tested whether Nile tilapia (N = 30) presented a DFE when handled (Figure 1 and Figure 2A,C—described in detail below), and all fish presented this response, each minute for 20 consecutive minutes (Appendix A). Fish were submitted to three stunning protocols: benzocaine anesthesia (positive control—two concentrations: 40 mg/L and 80 mg/L, N = 15), immersion in ice water (three temperatures ranges: 0–1 °C, 2–3 °C and 5–6 °C, N = 30) and CO_2_ stunning (N = 20). Although immersion into ice water and CO_2_ stunning are considered non-humane stunning protocols [12], we used these stunning methods since they are still the most used in Brazil and some European countries [5,14,15]. After the fish were submitted to one of these three stunning methods, we assessed the time for the VOR (Figure 2B,D) to be lost and its return, a clinical reflex and well-established indicator of unconsciousness/insensibility in fish [11,18,25], and the DFE, aiming to test whether this response to a stimulus presented in Nile tilapia (Appendix A) could be used as a visual sensibility indicator (VSI). To test the presence/absence of both VSI, we removed fish from the water using a dip net. The fish were not exposed to the air for more than 10 s to avoid any stress and painful stimuli for the fish [26]. To test the presence/absence of the VOR, we followed the protocol described by [18], and tested the presence/absence of the DFE (Figure 2A,C) by holding the fish with both hands and turning it 90° (Figure 1).

To apply the stunning protocols, fish were individually transferred to glass aquaria (20 L −40 × 23 × 25 cm^3^). For benzocaine stunning, we added benzocaine to the aquarium water in 40 mg/L or 80 mg/L concentrations. For stunning by immersion in ice water, we prepared a mixture of water and ice. We added ice until it reached the temperature ranges for the treatment (0–1 °C, 2–3 °C and 5–6 °C). The temperature was controlled by a thermometer, and when necessary, more ice was added to the water. Immediately afterwards, the fish were placed in an aquarium. We started to time how long it took for both VSIs to be lost. Each minute, we took fish from the water and tested the presence of the VOR and DFE (Figure 1). We stopped timing when both VSIs were absent. Immediately after the loss of both VSIs, the fish were transferred to aquariums of the same size, with continuous aeration and temperature of 25 °C, and we started to time how long both VSIs took to return. This procedure of timing how long the VSIs took to return was equal for all stunning protocols.

In CO_2_ stunning, fish were individually transferred to a 20 L aquarium (40 × 23 × 25 cm^3^) equipped with a thermometer and hose with porous stone connected to a CO_2_ cylinder. Using a different method to the other stunning protocols, fish VSIs were assessed each minute, defined as three different times to assess the fish DFE: 5, 8 and 10 min. We set these assessment times because 5 min before the amount of CO_2_ in the water was too low (Table S1) and did not cause any loss of VSIs. We bubbled CO_2_ into the water until near saturation. When fish lost both VSIs or when they remained in the water that was bubbled with CO_2_ for 10 min, they were transferred to the recovery aquaria. The water from the test aquarium was replaced between each fish to avoid CO_2_ accumulation. During the experiments, we collected water samples at the three times set to assess fish VSI to quantify the amount of free and total CO_2_ at these times (Table S1). These water samples were analyzed through titration with sulfuric acid, as described by [27].

### 2.3. Statistical Analysis

All statistical analyses were performed in the R environment (v3.6.0.) The Kaplan–Meier test was used to estimate the probability to the occurrence of the desired event over time (loss and return of VOR and DFE) by applying the survfit and ggsurvplot functions in R. For each stunning protocol, namely, benzocaine anesthesia and immersion in ice water, two Kaplan–Meier tests were performed for each VSI separately (VOR and DFE): one to compare the probability of losing the VSI over time between treatments of each stunning protocol (benzocaine anesthesia: 40 mg/L vs. 80 mg/L; immersion in ice water: 0–1 °C vs. 2–3 °C vs. 5–6 °C) and another to compare the probability of the VSI returning over time between treatments of each stunning protocol. For the CO_2_ stunning protocol, Kaplan–Meir tests were performed to compare the probability for the loss and return of different VSIs over time (VOR vs. DFE). Pairwise comparisons were performed using log-rank tests with the Bonferroni *p* value adjustment method by applying the pairwise_survdiff function in R. The significance level for all tests was set at α = 0.05.

## 3. Results and Discussion

**Figure 3:**
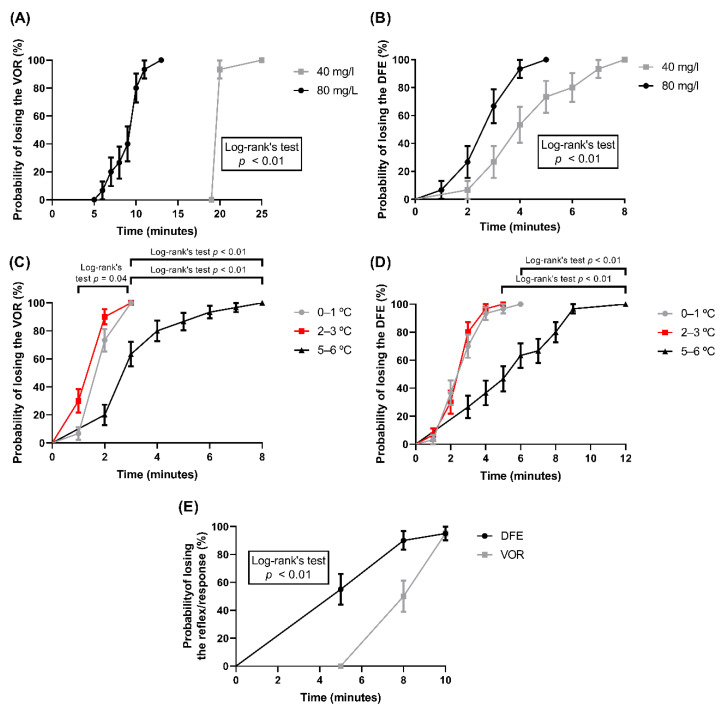
Kaplan–Meier survival curves of the probability of losing the reflexes and behaviors by different stunning protocols. (**A**) Probability of losing the VOR and the (**B**) DFE by benzocaine anesthesia (N = 15). (**C**) Probability of losing the VOR and the (**D**) DFE by immersion in ice water (N = 30). (**E**) Probability of losing the VOR and DFE by CO_2_ stunning (N = 20). Mean ± SE are shown. Statistical differences between the groups in log-rank tests are indicated in the graphs (*p* < 0.05).

In this study, the presence/absence of DFEs as a VSI in fish were investigated. Firstly, the efficiency of common stunning protocols was verified to induce the loss of a clinical reflex that is well-established to assess the state of fish sensibility, VOR [18,25]. All fish submitted to benzocaine anesthesia and immersion in ice water lost the VOR (Table 1, Figure 3A,C). CO_2_ stunning induced the loss of VOR in 95% of individuals. Regarding the DFE loss, similar results were observed, since 100% of fish stunned with benzocaine anesthesia and immersion in ice water lost this behavioral response, and 95% of individuals stunned with CO_2_ lost the DFE (Table 1, Figure 3B,D,E). Fish from all treatments showed a faster return of DFEs than VORs (Table 1 and Table 2). Therefore, the results showed that a DFE can be used as a VSI in Nile tilapia. However, the absence of a DFE alone does not ensure that fish have been totally stunned, and it must be used together with other well-established VSIs to obtain a more accurate assessment of the state of fish sensibility before slaughter in fish farms.

The VOR is a clinical reflex that has been well-established to assess the state of fish sensibility [11,18,25]. Usually, the VOR is lost in stage 4 of anesthesia, along with the responses to external stimuli and opercular beats decreasing [25]. All fish stunned with benzocaine and immersion in ice water lost the VOR (Table 1). The highest dose of benzocaine (80 mg/L) induced the VOR lost quicker (median = 10 min, Table 1) than the lower dose (40 mg/L), (*p* < 0.01, Figure 3A). Lower temperature ranges (0–1 °C and 2–3 °C) also induced the loss of VOR quicker (median = 2 min, Table 1) than the highest temperature range of 5–6 °C (*p* < 0.01, Figure 3C). Regarding the CO_2_ stunning protocol, 95% of fish lost the VOR, and the median time to lose the reflex was 9 min (Table 1; Figure 3E). The quick loss of VOR induced by immersion in ice water can be related to fish immobilization before insensibility, usually caused by this stunning method [19,28].

In addition to the VOR loss, some anesthetics in the stage 4 of anesthesia also induce the loss of responses to strong external stimuli, particularly behavioral responses to pressure on the caudal fin or peduncle (e.g., escape attempt on first pinch) [18,25]. Other responses to tactile external stimuli are lost in stage 3 of anesthesia [25,29]. A response to tactile stimulation present in some fish species (e.g., Nile tilapia) when they are handled is the DFE [21] that occurs without needing a painful stimulus. To verify whether the DFE was absent after handling (Figure 1 and Figure 2) anesthetized fish (positive control), and thus, check whether a response can be used as a VSI in stunning protocols applied at pre-slaughter production stage, we submitted fish to benzocaine anesthesia in two concentrations (40 mg/L and 80 mg/L). All fish submitted to benzocaine anesthesia lost the DFE. The highest dose induced a loss of the DFE quicker (median = 3 min, Table 1) than the lower dose (*p* < 0.01, Figure 3B). Stunning by immersion into ice water, a common stunning protocol for fish [12,17], also induced the loss of DFE in 100% of fish tested. Lower temperature ranges (0–1 °C and 2–3 °C) induced the loss of the DFE quicker (median = 3 min, Table 1) than a temperature range of 5–6 °C (*p* < 0.01, Figure 3D). Regarding CO_2_ stunning, this insensibility method induced the loss of the DFE in 95% of fish (Table 1), and the loss of the DFE was quicker in a higher percentage of fish than the loss of the VOR (*p* < 0.01, Figure 3E). The absence of clinical reflexes, such as the VOR and “breathing”, could already determine whether fish are insensible [18]. However, to ensure that a fish is completely insensible is difficult, and so, observing the presence/absence of responses to stimulus, such as the DFE, can provide a more accurate assessment, since fish are able to respond to a painless and low-intensity stimulus. They probably have sufficient brain function to perceive and suffer during the slaughter.

**Figure 4:**
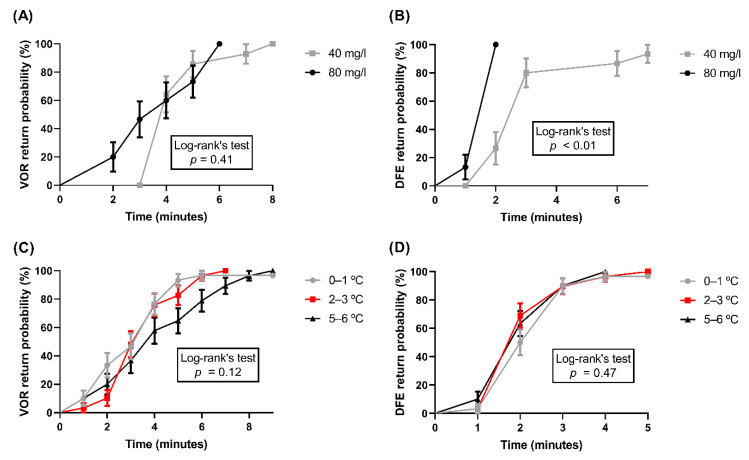

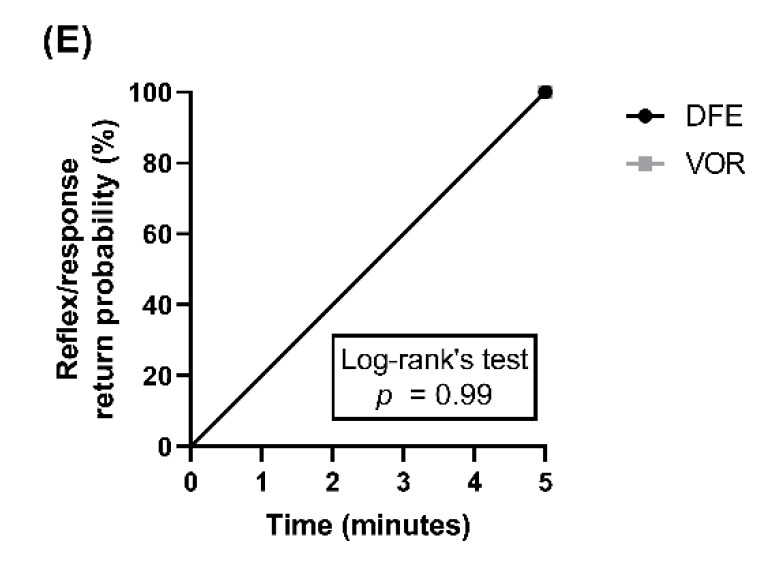
Kaplan–Meier survival curves of the probability that reflexes and behavior returns using different stunning protocols. (**A**) Probability of return of VOR and (**B**) DFE using benzocaine anesthesia (N = 15). (**C**) Probability of return of VOR and (**D**) DFE by immersion into ice water (N = 30). (**E**) Probability of return of VOR and DFE using CO_2_ stunning (N = 20). Mean ± SE are shown. Statistical differences between the groups in log-rank tests are indicated in the graphs (*p* < 0.05).

Regarding behavioral responses and the recovery of reflexes, these are usually the first overt signs that a fish has recovered after stunning [30]. Indeed, when fish are stunned, the first sign to return is the VOR [25]. However, our results show that in all experimental groups, DFE returned quicker than the VOR (Table 1 and Table 2; Figure 4). A DFE is characterized as a response to stimulus, since fish present the DFE in response to different stimulus, such as new stimuli (e.g., a tap on the aquarium lateral wall or a shadow moving overhead [31]), a threat (e.g., other conspecific or intraspecific disputes) or the presence of a predator [23], and when they are handled (Figure 1 and Figure 2A,B; Appendix A). However, our results regarding the return of the VSI suggest that maybe the DFE also could be a sensory reflex caused by external stimuli besides being a behavioral response caused by brain signals. Further studies can investigate the underlying neuro-mechanisms in fish DFE to determine whether it is indeed a response, a reflex or both. Besides this, the highest dose of benzocaine (80 mg/L) induced the DFE to return quicker (median = 2 min, Table 1) than the lower dose (40 mg/L) (*p* < 0.01, Figure 4B). The VSI that returns the quickest, such as the DFE, is important to be evaluated, given that once fish have presented a return of at least one VSI, they are able to perceive and react to any painful stimuli that are applied when they are stunned and/or unconscious [18].

## 4. Conclusions

Our results suggest that the DFE can be used as a VSI in fish. Assessing if the DFE is present/absent is easy, simple and quick for use in fish production. Moreover, the presence/absence of the DFE can be checked without applying painful stimuli to fish, which is different from other common responses to stimuli used to assess fish insensibility, such as a tail pinch, prick and shock on lip [11,18,32]. Assessing responses without the need for a painful stimulus to infer the state of fish sensibility has to be prioritized, since good welfare in fish needs to be free from fear and pain in all production stages, including the stunning, the assessment of insensibility and/or unconsciousness pre-slaughter and slaughter [11,28]. Another advantage of using the DFE as a VSI is their fast recovery. VSIs that return first are important to be evaluated so that fish do not suffer. It is important that they do not present VSIs immediately before slaughter. However, the absence of the DFE alone does not ensure that fish are totally insensible, so it must to be used together with other painless VSIs that are well-established for fish and the Nile tilapia, such as the VOR, opercular beats and self-initiated behavior (e.g., swimming and equilibrium) [11,18]. Further studies must evaluate the efficiency of the DFE as a VSI in other fish species and using other stunning protocols, such as electrical and percussive stunning methods considered humane for fish stunning [12].

## Figures and Tables

**Table 1:** Data summary table. Time for the loss and return (range and median) of the VOR and DFE and the percentage of fish where these visual stunning indicators were lost and returned, following the different stunning protocols.

Stunning Protocol	VOR ^1^						DFE ^2^					
	Lost			Return			Lost			Return		
	Range (min)	Median (min)	Fish (%)	Range (min)	Median (min)	Fish (%)	Range (min)	Median (min)	Fish (%)	Range (min)	Median (min)	Fish (%)
Benzocaine (40 mg/L)	20–25	20	100	0–8	4	100	2–8	4	100	0–7	3	93.33
Benzocaine (80 mg/L)	6–13	10	100	2–6	4	100	1–5	3	100	1–2	2	100
Ice water (0–1 °C)	1–3	2	100	1–6	4	96.67	1–6	3	100	1–4	2.5	96.67
Ice water (2–3 °C)	1–3	2	100	1–7	4	96.67	1–5	3	100	1–5	2	96.67
Ice water (5–6 °C)	2–8	3	100	2–9	4	96.67	3–12	6	100	1–4	2	96.67
CO_2_ stunning	8–10	9	95	5–5	5	100	5–10	5	95	5–5	5	100

^1^ VOR—Vestibulo-ocular-reflex. ^2^ DFE—Dorsal Fin Erection.

**Table 2:** Percentage of fish that lost and recovered none/only one/both VSIs and percentage of fish that lost and recovered DFEs before VORs and vice versa.

Stunning Protocol	Lost					Return						
	None VSI ^1^ (%)	Only One VSI (%)	Both VSI (%)	VOR ^2^ before DFE ^3^ (%)	DFE before VOR (%)	Both VSI at Same Time (%)	None VSI (%)	Only One VSI (%)	Both VSI (%)	VOR before DFE (%)	DFE before VOR (%)	Both VSI at Same Time (%)
Benzocaine (40 mg/L)	0	0	100	0	100	0	0	0	100	0	93.33	6.67
Benzocaine (80 mg/L)	0	0	100	0	100	0	0	0	100	0	86.67	13.33
Ice water (0–1 °C)	0	0	100	66.67	6.67	26.67	0	0	100	10.35	58.62	31.03
Ice water (2–3 °C)	0	0	100	80	3.3	16.67	0	0	100	3.45	86.21	10.34
Ice water (5–6 °C)	0	0	100	83.33	0	16.67	0	0	100	3.45	79.31	17.24
CO_2_ stunning	5	0	95	0	90	10	0	0	100	0	0	100

^1^ VSI—Visual sensibility indicator. ^2^ VOR—Vestibulo-ocular-reflex. ^3^ DFE—Dorsal Fin Erection.

## Data Availability

The data presented in this study are available on request from the corresponding author.

## Supplementary Materials

The following are available online at https://www.mdpi.com/article/10.3390/ani11103007/s1, Table S1: Water saturation by free and total CO_2_ over time.

## Appendix A

### Fish Not Submitted to Any Stunning Protocol-DFE and VOR Presence Control

We verified whether Nile tilapia (N = 30) presents both visual sensibility indicators assessed in this study, the VOR and the DFE, when not submitted to any stunning protocol. Fish were individually transferred to glass aquaria (20 L −40 × 23 × 25 cm^3^) with a water temperature of 25 ± 1 °C and constant aeration. Immediately after fish were placed into the aquarium, we started timing, and at every minute, for 20 consecutive minutes, we removed fish from the water and tested the presence or absence of both visual sensibility indicators. The time that fish were exposed to air was less than 10 s. The VOR was assessed following the protocol described by [18], and to verify DFE presence, we held the fish in both hands and turned them 90° (Figure 1). All fish that were not submitted to any stunning protocol (N = 30) presented both visual stunning indicators, the VOR and the DFE, for each minute, over 20 consecutive minutes.
