# Supplementary material for: Welfare in Nile Tilapia Production: Dorsal Fin Erection as a Visual Indicator for Insensibility
Source: Animals (Basel). 2021 Oct 19;11(10):3007. doi: 10.3390/ani11103007 (PMC8532844; doi:10.3390/ani11103007)
Supplement: Supplementary file 1 [file animals-11-03007-s001.zip › animals-1376452-supplementary.pdf]

Supplementary Materials

Table S1. Water saturation by free and total CO_2 over time.

Time (min)	0	5	8	10
**Total CO_2 (mg/L)**	2.11 ± 1.17	24.69	39.25	40.32
**Free CO_2 (mg/L)**	0.48 ± 0.12	22.57	36.45	39.32
